# Comparison of illegal drug use pattern in Taiwan and Korea from 2006 to 2014

**DOI:** 10.1186/s13011-016-0078-x

**Published:** 2016-09-23

**Authors:** Ling-Yi Feng, Wen-Jing Yu, Wei-Ting Chang, Eunyoung Han, Heesun Chung, Jih-Heng Li

**Affiliations:** 1 School of Pharmacy and Ph.D. Program in Toxicology, College of Pharmacy, Kaohsiung Medical University, 100 Shih-Chuan 1st Road, Kaohsiung City, 80708 Taiwan; 2 College of Pharmacy, Duksung Women’s University, Seoul, Korea; 3 Graduate School of Analytical Science and Technology(GRAST), Chungnam National University, 99- Daehak-ro, Yuseongk-gu, Daejeon, 305-764 Korea

**Keywords:** New Psychoactive Substances (NPS), Drug seizures, Ketamine, Methamphetamine, Taiwan, Korea

## Abstract

**Background:**

Illegal drug use has long been a global concern. Taiwan and Korea are geographically adjacent and both countries have experienced the illegal use problems of methamphetamine, a predominant prototype of New Psychoactive Substances (NPS). NPS, a term coined by the United Nations Office on Drugs and Crime (UNODC) in recent years, have not been scrutinized for their safety and may become a new threat to public health and security worldwide. To conduct evidence-based drug policy, it is imperative to estimate the trend and pattern of illegal drug use. Therefore, this study aims to analyze and compare the current status of drug-related seizures, arrests and illegal drug use, with a focus on methamphetamine and NPS, between Taiwan and Korea.

**Methods:**

Data of illegal drug (including NPS)-related seizures and arrests were collected via anti-drug related agencies of both countries from 2006 through 2014.Since listing of NPS as controlled substances was a result of NPS abuse liability through official evaluation, the items of controlled NPS were used as an indicator of emerging use. These data obtained from Taiwan and Korea was then compared.

**Results:**

The results showed that while methamphetamine remained as a predominant drug in both Taiwan and Korea for decades, different illegal drug use patterns have been observed in these two countries. In Taiwan, the major illegal drugs were methamphetamine, heroin, and ketamine, whereas in Korea those were methamphetamine and cannabis. By comparison of *per capita* illicit drug seizures, the illegal drug use situation in Taiwan was at a higher stake than that in Korea. In terms of NPS use, ketamine has been a major drug in Taiwan, but it was seldom found in Korea. Besides ketamine, the major type of NPS was synthetic cathinones in Taiwan whereas it was synthetic cannabinoids and phenethylamines in Korea. The difference in the numbers of controlled NPS items between Taiwan (23) and Korea (93) may be due to the implementation of temporary control on NPS in Korea since 2011.

**Conclusion:**

While the problem of methamphetamine still lingers, NPS have emerged as a new issue in both countries. However, the NPS pattern was different between Taiwan and Korea. Although the controlled NPS items in Taiwan were far less than those in Korea, the quantity of total NPS seizures, especially with ketamine, was much larger in Taiwan than in Korea. Different NPS pattern may also imply they were from different sources. Factors other than geographical proximity, such as drug policy and availability and accessibility to drugs, should be taken into account for the current status of illegal drug use in Korea and Taiwan.

## Background

Humans have experienced a long history of drug (or substance) use. To tackle the profound drug-related issue, the United Nations has promulgated three international anti-drug conventions in the twentieth century, namely, the 1961 Single Convention on Narcotic Drugs, the 1971 Convention on Psychotropic Substances, and the 1988 Convention against Illicit Traffic in Narcotic Drugs and Psychotropic Substances [1–3]. These three anti-drug Conventions provide legal mechanisms for the control of narcotics, psychotropic substances and precursors. According to the 2015 World Drug Report of United Nations Office on Drugs and Crime (UNODC), cannabis, opioids/opiates and amphetamine-type stimulants (ATS) are currently the top three illicit drugs worldwide [4]. However, reviews on the major illicit drug use situation in some Asian countries, such as China, India, Japan, Malaysia, Taiwan, and Vietnam, showed that although these three illicit drugs have been most prevalent in general, ATS is not a favorable drug in India, organic solvent replaces opioids/opiates as one of the top three illicit drugs in Japan, and ketamine replaces cannabis in both China and Taiwan [5–7]. These studies indicate that the status of illegal drug use may vary from one country to another. Thus, evaluation of illegal drug use situation at individual country level is necessary to solve unique drug problems in each country.

In recent years, the UNODC has warned the emergence of new psychoactive substances (NPS) [8, 9]. NPS are classified by the UNODC as synthetic cannabinoids, synthetic cathinones, ketamine and PCP-type substances, phenethylamines, piperazines, tryptamines, aminoindanes, plant-based substances and others [10]. They are not only dangerous to individual health but also intimidating to public health and social security due to their uncertain toxicological profiles [11–13]. The NPS use has become a new global challenge because they are predominantly derivatives or analogues of existing controlled substances and remain mostly elusive from the UN Conventions.

In Taiwan, heroin and methamphetamine have been the predominant illicit drugs since 1990s. HIV infection by needle/solution sharing among heroin injecting users surged in the early 2000s but was contained within a decade after implementation of harm reduction measures [11]. Methamphetamine, a schedule II substance listed in the 1971 Convention on Psychotropic Substances, was originally a pharmaceutical that was legally manufactured but widely misused in Japan after World War II. Illegal use of methamphetamine then spread to Republic of Korea (a.k.a South Korea, hereby abbreviated as Korea) and Taiwan in the 1970s and early 1990s, respectively. People in both countries have witnessed the methamphetamine epidemic since the late 20th Century [12]. In Taiwan, while the problems of methamphetamine and heroin still lingering, other major illicit drugs such as ketamine and MDMA have emerged since the past decade [5, 14, 15]. In addition, illegal use of NPS other than ketamine, such as some synthetic cannabinoids [e.g., JWH-250(K2), JWH-018(K2)], synthetic cathinones [e.g., mephedrone, MDPV (methylenedioxypyrovalerone)] and Salvia (*Salvia divinorum*) has also been reported [13, 16, 17]. Hence, illicit drugs consumed in Taiwan include not only the items in the 1961 Convention such as heroin, those in the 1971 Convention such as methamphetamine and MDMA, but also the NPS items such as ketamine, synthetic cannabinoids and cathinones.

In Korea, the top three illicit drugs were methamphetamine, cannabis and opiates in 2004 [18]. Synthetic drugs such as MDMA, Yaba, and LSD were found in greater proportion in the seizure records. The misuse of common medicines, such as dextromethorphan, zipeprol, and carisoprodol, was also found among young people because of their easy availability. In recent years, seizures of NPS, such as the synthetic cannabinoids JWH-018 or the plant-based substance kratom, have been reported in Korea. Synthetic cannabinoids (JWH-018 and its analogues), first detected in 2008 [19], have been identified as an emerging threat in Korea. Synthetic cannabinoids accounted for 71 % of total confiscated new drugs, followed by 18 % of phenethylamines, 7 % of piperazines and4% of tryptamines [20]. Traditional drugs, including heroin and cocaine, are not commonly used in Korea as reflected by drug seizure and arrest data [19]. Therefore, the illegal drug use situation may have changed over the last decade in Korea. However, comprehensive and updated information has not yet been available.

Methamphetamine, a member of the phenethylamine family, can be regarded as a prototype of NPS. Since both Taiwan and Korea have been the victims of methamphetamine use problems for decades, the outcomes of their drug policy and countermeasures have not been thoroughly approached. Illegal ketamine use has recently become a serious problem in Taiwan. However, the illegal drug use data has not been incorporated into the reports of the United Nations because Taiwan is currently not a member of the UN. According to the latest annual report of Taiwan Food and Drug Administration (TFDA), the amounts of ketamine seizure increased sharply in Taiwan in recent years [21] and the sources of ketamine mainly originated from China and India. Ketamine seized in Indonesia and Japan was perceived to originate from China and/or India between 2008 and 2012 [22]. Since Korea is geographically adjacent to China, Taiwan and Japan, it would also be of interest to explore if Korea is currently under the threat of illicit ketamine use. In addition, many NPS items have been identified in both Taiwan and Korea, it is also worthy of comparing the NPS problems between the two countries.

With comparable economic status, similar historic background and the same methamphetamine problems, it would be intriguing to compare the progress of methamphetamine problems and the emerging NPS issue between these two countries over the years. Drug seizures and arrests, which are usually the direct results of drug law enforcement (DLE), are often viewed as the most important purpose of DLE [23]. However, it is arguable that drug seizures and arrests are indicators of the presence of illegal drugs and illegal activity, not of drug use. But for countries like Taiwan and Korea, all illegal drug activities, including trafficking/smuggling, manufacturing, possession and use, are regarded as criminal offenses. Therefore, in this paper, we tried to analyze the data of drug-related criminal offenses. Through sorting illegal drug use from possession, manufacturing and trafficking/smuggling, the activity of illegal drug use could be interpreted as, at least in part, of illegal drug use situation. Based on the comparable data obtained from Taiwan and Korea, we would also like to learn if the experiences obtained from tackling the methamphetamine problems could be applied to the NPS issue.

## Methods

### Data sources

The trend and pattern of substance use in Taiwan are monitored via the national substance use detection and reporting system, which is composed of: (1) a subsystem of data collection on arrests, seizures and laboratory testing for urine samples. These data are gathered from several agencies including Taiwan Food and Drug Administration (TFDA), Ministry of Health and Welfare; the National Police Administration, Ministry of Interior; and Department of Health; (2) a subsystem of reporting for addiction treatment; The data are collected from the Ministry of Health and Welfare-designated hospitals with psychiatry specialty; (3) National household survey: the survey are performed every 5 years by the Ministry of Health and Welfare to explore the lifetime prevalence of substance use in the general population [24]. Based on the data from these subsystems, the TFDA publishes the statistical annual reports on the website (http://www.fda.gov.tw/TC/site.aspx?sid=1578).

In Korea, the drug misuse or illegal use monitoring system has been established to collect data from Supreme Prosecutors’ Office (SPO), the National Forensic Service (NFS), Korea Customs Service (KCS), and Korean Association Against Drug Abuse (KAADA). The KAADA has been designated to integrate data and publish white papers on drug-related crimes yearly since 2000 (http://www.spo.go.kr/eng/division/statistics/statistics.jsp) [25].

In both countries, listing of NPS as controlled substances was a result of NPS abuse liability through official evaluation. Therefore, the items of controlled NPS were used as an indicator of emerging use.

In this study, the drug-related data were collected from these official systems in Taiwan and Korea from 2006 to 2014.

### Drug scheduling information and analysis of NPS controlled items

In Taiwan, illicit drugs are classified into schedule I to VI according to their potentials of addiction, abuse, and harms to society. In Korea, the illicit drugs are classified as narcotic drugs, psychotropic agents, cannabis, and precursor chemicals based on the Act on the Control of Narcotics. Table 1 illustrated the difference in drug classification/schedules between Taiwan and Korea. Because newly listed items of NPS imply that there have been evidence of NPS use, these NPS items were collected and compared between the two countries.

**Table 1:** Different drug classification/schedules in Taiwan and Korea

	Taiwan	Korea
Schedule/classification rule	The drugs are classified into four schedules according to their potentials of addiction, abuse, and harm to society	1. Narcotic Drugs (3 categories) 2. Psychotropic Agents (4 schedules) 3. Cannabis 4. Precursor chemicals(2 categories)
Examples	1. Schedule I: heroin, morphine, cocaine, opium 2. Schedule II: amphetamine, cannabis, methamphetamine, MDMA, methadone 3. Schedule III: flunitrazepam, ketamine 4. Schedule IV: lorazepam、zolpidem	1. Narcotic Drugs: (1) I (natural narcotic): opium (2) II (alkaloid):morphine, heroin, cocaine (3) III (synthetic chemical): methadone 2. Psychotropic: (1) I:methamphetamine (2) II: ketamine, amphetamine (3) III: Thiopental (4) IV: Propofol 3. Cannabis 4. Precursor chemicals: I: Ephedrine II: Piperidine

### Data analysis

Data were collected and analyzed by descriptive statistics in this study. The trends and patterns of illicit drug seizures between Taiwan and Korea were compared. The main indicators, including the amounts of major drug seizures, *per capita* seizure and drug arrests, were adopted for comparison.

## Results

### Comparison of illegal drug use situation between Taiwan and Korea

#### Major illicit drugs

To evaluate the illegal drug use situation, the items and amounts of drug seizures and the numbers of drug-related arrests were utilized to provide an estimate. The amounts of drug seizures from 2006 through 2014 in Taiwan and Korea are shown in Tables 2 and 3, respectively. In the order of seizure amounts, the major illicit drugs were ketamine, methamphetamine, and heroin in Taiwan, while those were methamphetamine and cannabis/marijuana in Korea. In Taiwan, besides methamphetamine, it is noteworthy that there was an escalating increase in ketamine seizure from 2006 to 2014 along with the appearance of other NPS since 2013 (Table 2). In Korea, as shown in Table 3, although the expression of units was somewhat different among drugs, it can still be identified that methamphetamine remained at the highest seizure amounts from 2006 to 2014 whereas marijuana/cannabis was popular before 2012. JWH-018 and its analogs emerged as the major NPS group since 2009. Illicit use of propofol has become popular in recent years. Heroin has been a major drug problem in Taiwan. In contrast, the heroin seizure was almost negligible in Korea. However, the seizure of poppy plants in large quantities was only reported in Korea, implying the growth of opium poppy in situ or nearby (Table 3).

**Table 2:** Seizure amounts of major illicit drugs in Taiwan from 2006 to 2014

Category	Illicit Drug	Unit	2006	2007	2008	2009	2010	2011	2012	2013	2014
Schedule I	Heroin	Kg	203.48	137.67	130.52	62.42	83.61	17.84	157.94	104.1	86.74
Schedule II	Methamphetamine	Kg	181.37	124.33	28.37	107.02	251.86	140.6	119.3	775.85	462.93
	Cannabis	Kg	28.04	22.32	13.21	61.07	21.01	1.589	14.35	35.75	10.73
Schedule III	Ketamine	Kg	827.9	598.7	799.5	1186.4	2594.3	1371.9	2111.1	2393.3	3303.2
NPS	Others	Kg	0	0	0	0	0	0	0	16.39	30.64

**Table 3:** Seizure amounts of major illicit drugs in Korea from 2006 to 2014

Category	Illicit Drug	Unit	2006	2007	2008	2009	2010	2011	2012	2013	2014
Narcotic	Poppy	Stump	32,081	37,275	35,488	113,422	38,554	37,443	22,753	25,369	65,023
	Raw poppy	Kg	0.098	0.137	0.395	0.166	0.05	-	-	-	0.11
	Heroin	Kg	0.018	-	-	1.914	0.081	-	0.004	-	-
	Cocaine	Kg	4.772	0.079	8.869	0.298	-	2.153	0.064	1.215	0.011
Psychotropic	Methamphetamine	Kg	21.543	23.739	25.572	15.189	11.888	23.466	20.716	37.689	47.680
	MDMA	Kg	0.356	18.323	0.236	0.295	0.16	0.185	0.774	0.407	0.216
	YABA	Kg	-	0.196	0.151	0	0.002	0.002	0.133	1.319	0.93
	LSD	Kg	-	-	-	-	-	-	0.011	-	0.008
	JWH-018 & Analog	Kg			-	0.063	0.194	1.183	4.454	1.107	0.049
	Propofol	Ampoule (50 ml)	-	-	-	-	-	2,004	20,202	159	319
	Others	Kg				4.449	4.789	3.840	9.264	10.172	15.017
Cannabis	Cannabis	Stump	3,890	4,251	3,385	12,690	3,244	70,916	5,195	8,072	5,088
	Marijuana	Kg	20.859	22.202	92.692	122.539	44.484	83.559	21.722	24.396	23.315
	Seed	Kg	62.186	10.684	61.196	218.156	37.048	28.229	27.871	6.215	4.391
	Hashish	Kg	0.158	0.761	2.021	0.517	0.038	0.06	0.334	0.066	0.334

**Fig. 1:**
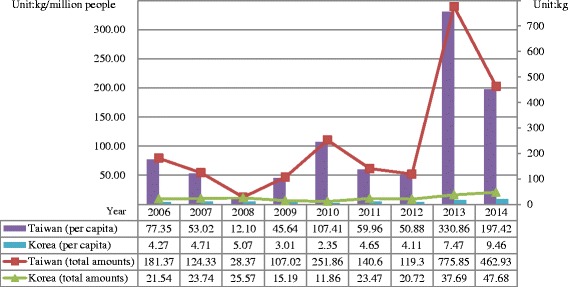
Total and *per capita* amounts of methamphetamine seizure in Taiwan and Korea from 2006 to 2014. The trend of methamphetamine seizure was upward in both Taiwan and Korea but the stake seemed to be higher in Taiwan

Therefore, in both Taiwan and Korea, methamphetamine that has long been a predominant illicit drug still remains as a major drug. The total amounts of methamphetamine seizure in Taiwan increased from 181.37 kg in 2006 to 462.93 kg in 2014 with a peak of 775.85 kg in 2013, and increased from 21.54 kg in 2006 to 47.68 kg in 2014 in Korea (Fig. 1). Although both countries have suffered from the deluge of illegal methamphetamine use for decades, the amounts of seizure in Taiwan, in total or *per capita*, are larger than those in Korea (Fig. 1).

#### NPS

**Fig. 2:**
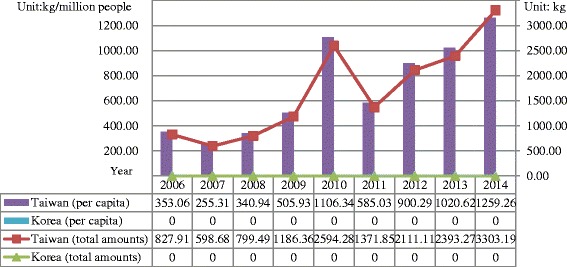
Total and *per capita* amounts of ketamine seizure in Taiwan and Korea from 2006 to 2014

**Fig. 3:**
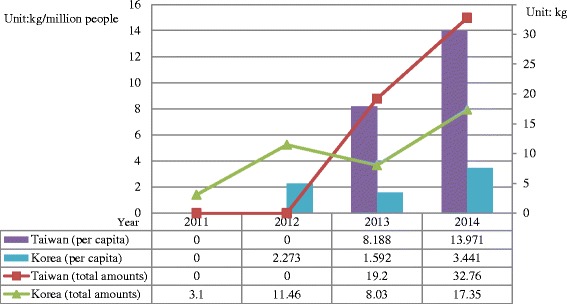
Total and *per capita* amounts of NPS (excluding ketamine) seizures in Taiwan and Korea from 2006 to 2014

Ketamine has been the primary NPS in Taiwan since 2006. The amounts of ketamine seizure in Taiwan increased yearly from 828 kg in 2006 to 3,303 kg in 2014but no ketamine confiscation was reported in Korea (Fig. 2). Other than ketamine, synthetic cannabinoids (JWH-018 and analogues) were more popular in Korea, whereas synthetic cathinones (MDPV, 4-MMC, bk-MDMA) and XLR-11 were the main NPS in Taiwan. The seized amounts of NPS(excluding ketamine), increased from 0 kg in 2012 to 32.76 kg in 2014 in Taiwan, and a similar increasing trend from 3.1 kg in 2012 to 17.35 kg in 2014 in Korea (Fig. 3).

According to the report of UNODC, most NPS have not been controlled by the 1961 Single Convention on Narcotic Drugs or the 1971 Convention on Psychotropic Substances, but they have been linked to health problems [26]. A recent World Drug Report noted 540 different NPS had been identified [4]. In order to control the spread of NPS, many countries have regulated these substances under different national legislations in succession. Due to the rapid and easy modification of the chemical structures and the continuous emergence of new substances, it is hard pressed to comprehensively regulate all emerging NPS. In accordance with the data that were collected from public sectors in Taiwan and Korea, some NPS items have been controlled, reflecting the fact that these NPS items have been illegally used in either country (Table 4).The difference in the numbers of controlled NPS items between Taiwan (23) and Korea (93) may be due to the temporary scheduling system that was added to the Korean Act on the Control of Narcotics in 2011, resulting in a surge of listed NPS items in Korea from then on [24]. Under the Act, the Korean Food & Drug Administration may temporarily schedule NPS for a year. The synthetic cathinone MDPV (3,4-Methylenedioxypyrovalerone) was the first drug subject to temporary schedule at the end of 2011.

**Table 4:** Controlled NPS in Taiwan and Korea by year

Year	Taiwan	Category(Number)	Korea	Category(Number)
~2008	N,N-Dimethylamphetamine- (DMA) / 2,5-Dimethoxyamphetamine p-Methoxymethamphetamine (PMMA)	Phenethylamines (2)	5-MeO-DiPT	Tryptamines (1)
2009	p-Methoxyethylamphetamine (PMEA)	Phenethylamines (1)	JWH-018, HU-210, CP-47,497, 4-methylmethcathinone, 5-MeO-MiPT, 5-MeO-AMT, 4-Acetoxy-DiPT	Synthetic cannabinoids (3) Synthetic cathinones (1) Tryptamines (3)
2010	Mephedrone (4-MMC)	Synthetic cathinones (1)	5-MeO-DMT	Tryptamines (1)
2011	CP-47,497, HU-210, JWH-018, JWH-073JWH-250, 5-MeO-DIPT	Synthetic cannabinoids (5) Tryptamines (1)	Analogs of JWH-018 (naphthoylindoles), CP-47,497, methcathinone, and phencyclidine, MDPV	Ketamine and phencyclidine-type substances (1) Synthetic cannabinoids (2) Synthetic cathinones (2)
2012	MDPV (3,4-Methylenedioxypyrovalerone), Methylone (bk-MDMA),Ketmine, 2-Fluoromethamphetamine (2-FMA), 3-Fluoromethamphetamine (3-FMA), 4-Fluoromethamphetamine (4-FMA), TFMPP	Ketamine and phencyclidine-type substances (1) Phenethylamines (4) Piperazines (1) Synthetic cathinones (1)	4-Fluoroamphetamine and 4-methylamphetamine	Phenethylamines (2)
2013	AM-2201, JWH-122	Synthetic cannabinoids (2)	6-APB(Benzo Fury), methiopropamine, 5-MAPB, 5-APDB(EMA-4, 3-Desoxy-MDA), α-methyltryptamine(αMT, AMT, Indopan), p-chloroamphetamine(PCA, 4-CA), NMT, AB-001, ADB-FUBINACA, ADBICA, AB-PINACA, QUPIC(PB-22), 4-HO-DET(CZ-74, ethocin), 2,3-DCPP, Desoxy-D2PM(A3A, Methano, Green powder), JWH-030, α-PVT, JWH-307, 5-Fluoropentyl-3-pyridinoylindole, MDAI, AM-1241, and 5 F-PB-22,25I-NBOMe, 2C-C-NBOMe, 3-Fluoromethamphetamine, 5-(2-Aminopropyl)indole, 5-IAI, Dimethoxy-methamphetamine, Dimethylamphetamine, DOC, Ethylphenidate, Lisdexamphetamine, Phenazepam, MT-45, 4-AcO-DiPT, 5-MeO-EPT, 5 F-NNEI, A-834,735, AB-FUBINACA, NNEI, QUCHIC, RCS-4 ortho-isomer, AH-7921, alkyl nitrite(isobutyl nitrite, isopropyl nitrite, pentyl nitrite, isopentyl nitrite, tertiarybutyl nitrite, cyclohexyl nitrite, and butyl nitrite)	Aminoindanes (2) Other substances (1) Phenethylamines (17) Piperazines (2) Synthetic cannabinoids (16) Synthetic cathinones (1) Tryptamines (5)
2014	XLR-11, 3-Fluoromethcathinone (3-FMC), 4-Fluoromethcathinone (4-FMC), 25B-NBOMe (2C-B-NBOMe)	Phenethylamines (2) Synthetic cannabinoids (1) Synthetic cathinones (1)	MN-18, 5 F-MN-18, Methyl-1-(cyclohexylmethyl)-1H-indole-3-carboxylate, 5 F-AB-PINACA, FUB-PB-22, 5 F-ADBICA, A-836339, p-Chloromethamphetamine, p-Bromoamphetamine, 25B-NBOMe, 25D-NBOMe, 25H-NBOMe, 5-EAPB, 2C-C, 2C-P, N-methyl-2-AI, 3,4-dichloromethylphenidate, W-15, RH-34, N-ethyl-norketamine, Mepirapim, XLR-12, ADB-PINACA, FDU-PB-22, AB-CHMINACA, 5 F-AMB, 2C-N, βk-2C-B, acetylfentanyl, LY2183240, Revise rules in detail, add list (JWH-030, JWH-175, JWH-176)	Aminoindanes (1) Other substances (3) Phenethylamines (12) Synthetic cannabinoids (17)
Sum	23	Ketamine and phencyclidine-type substances (1) Phenethylamines (9) Piperazines (1) Synthetic cannabinoids (8) Synthetic cathinones (3) Tryptamines (1)	93	Aminoindanes (3) Ketamine and phencyclidine-type substances (1) Other substances (4) Phenethylamines (31) Piperazines (2) Synthetic cannabinoids (38) Synthetic cathinones (4) Tryptamines(10)

In Taiwan, 9 phenethylamines with stimulant or psychedelic effects have been identified and listed as controlled drugs since 2008. In 2010, mephedrone was controlled due to its similar chemical structure with cathinone and similar effects to MDMA, amphetamines and cocaine. Two other synthetic cathinones and 8 synthetic cannabinoids were sequentially identified and controlled. In Korea, the synthetic cannabinoids, with 38 items being controlled between 2009 and 2012, topped the NPS control list. The reported cases of synthetic cathinones, such as MDPV, also dramatically increased in 2011. Thirty-one items of phenethylamines and other types of NPS have also been regulated since 2014. These results indicate that the categories of NPS identified in Korea have been diversified with a majority of synthetic cannabinoids and phenethylamines.

From the results of drug seizures (supply side) and drug-related arrestees (illegal drug users stands for a proportion of demand side), it seems the overall illicit drug problem in Taiwan was more severe than that in Korea. The major illicit drugs in Taiwan were heroin, methamphetamine, and ketamine; in Korea, they were methamphetamine and cannabis. The trend of all illicit drug seizures went up slightly from 2006 to 2014, but the trend of drug-related arrests seems to be flat in recent years. Moreover, the emerging NPS problem has made the illegal drug use patterns become more diversified in both Taiwan and Korea.

### Comparison of drug-related arrests between Taiwan and Korea

**Fig. 4:**
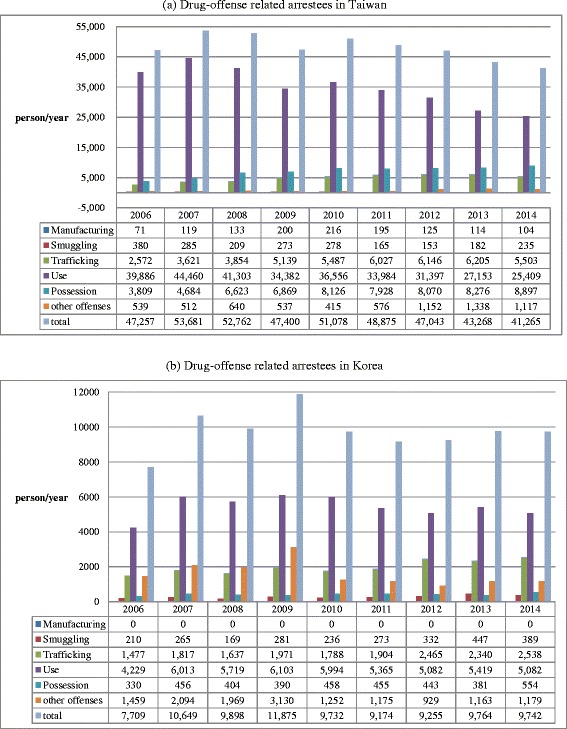
Comparison of drug-related arrests between Taiwan and Korea from 2006 to 2014. Most of the drug-related arrests were due to illegal drug use, which is a criminal offense in both Taiwan and Korea. **a** Drug-offense related arrestees in Taiwan. **b** Drug-offense related arrestees in Korea

**Fig. 5:**
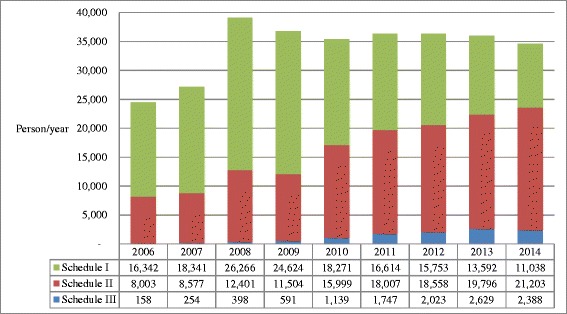
Number and proportion of drug-offense related arrestees according to drug types in Taiwan from 2006 to 2014. Heroin was the major drug in Schedule I, methamphetamine in Schedule II, and ketamine in Schedule III

**Fig. 6:**
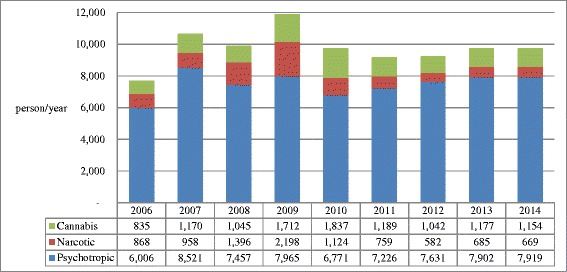
Number and proportion of drug-offense related arrestees according to drug schedules in Korea from 2006 to 2014. Methamphetamine was the major psychotropic agent

In Figs. 4, 5, and 6 the results of drug-related arrests in Taiwan and Korea from 2006 through 2014 are demonstrated. Since illegal drug use is a criminal offense in both Taiwan and Korea (Table 5), the data on drug-related arrests may represent, at least a proportion of the drug users in the demand side. In Fig. 4, an average of 34948 (72.7 %) and 5445 (55.8 %) of the drug-related arrestees were illicit drug users in Taiwan and Korea, respectively, representing a majority of arrestees were illegal drug users. The numbers of illegal drug use arrestees in Taiwan increased quickly from 39,886 people in 2006 to 44,460 people in 2007, and then gradually decreased to 31,397 in 2014 (Fig. 4a). In Korea, the numbers increased from 7,709 people in 2006 to 11,875 people in 2009, then stabilized at ca. 10,000 people till 2014 (Fig. 4b). Put the data of drug-related arrests and those of seizures together, the illegal drug use situation in Taiwan was more worrisome than that in Korea. The results of drug-related arrests in Taiwan (Fig. 5) showed that methamphetamine (scheduling II), heroin (schedule I) and ketamine (schedule III), were the three major illegal drugs. The decrease in illegal drug users over the years in Taiwan was mainly due to the reduction of illegal heroin users. Along with the results of drug seizures (Table 2), the top three most used illicit drugs in Taiwan were indeed heroin, ATS (mainly methamphetamine), and ketamine, which coincide with a latest study [5]. By comparison, in Korea, most of the drug-offense related arrestees were associated with methamphetamine, which was classified as a psychotropic substance (Fig. 6). Linking with the data of drug seizures in the supply side (Table 3), it could be deduced that the primary illicit drug in Korea was still methamphetamine.

**Table 5:** Comparison of drug-related laws between Taiwan and Korea

	Taiwan	Korea
Authorities	Ministry of Justice	Ministry of Justice
Laws	Statute for the Prevention and Control of Illicit Drugs	Act on the Control of Narcotics
Definition	Schedule I to VI	1. Narcotic Drugs 2. PsychotropicAgents 3. Cannabis 4. Precursor chemicals
Punishment	1. Illicit use ◆ Schedule I drugs:imprisonment for six months to five years ◆ Schedule II drugs: imprisonment for not more than three years 2. Possession ◆ Schedule I drugs: imprisonment for not more than three years ◆ Schedule II drugs:imprisonment for not more than two years ◆ Schedule III drugs over 20 g(net): imprisonment for not more than one years ◆ Schedule VI drugs over 20 g(net): imprisonment for not more than three years 3. Smuggling and manufacturing ◆ Schedule I drugs: imprisonment for deathor life sentence ◆ Schedule II drugs: imprisonment for life sentence or more than seven years ◆ Schedule III drugs: imprisonment for more than seven years ◆ Schedule VI drugs: imprisonment for five to twelve years	1. Illicit use ◆ Narcotic drugs:imprisonment for not more than ten years ◆ Psychotropic Agents: imprisonment for not more than ten years ◆ Cannabis: imprisonment for not more than five years 2. Possession ◆ Narcotic drugs:imprisonment for life sentence or not less than five years ◆ Psychotropic Agents: imprisonment for life sentence, or not less than five to ten years ◆ Cannabis: imprisonment for life sentence or not less than five years 3. Smuggling and manufacturing ◆ Narcotic drugs:imprisonment for life sentence or not less than five years ◆ Psychotropic Agents: imprisonment for life sentence or not less than five years ◆ Cannabis: imprisonment for life sentence or not less than five years ◆ Precursor chemicals: imprisonment for not more than five years

### Drug-related legislations and policy in Taiwan and Korea

Both Taiwan and Korea have very strict laws on illicit drugs (Table 5). It would result in long jail sentences and large fines for possession, use, or trafficking/smuggling of illicit drugs. Since July 1, 2000, Korean Act on the Control of Narcotics further incorporated the former Cannabis Control Act, Narcotics Act and the Psychotropic Substances Control Act to tackle the narcotics issue. Korean society pays less attention to illegal drug use and related social problems because it has been called ‘the country free from the needle’ with low levels of narcotics misuse [27]. In Korea, drug addicts are treated by the Ministry of Health and Welfare Affairs at 22 hospitals nationwide. The treatment is free and addicts can stay in the program for one year [28].

Taiwan was excluded from the UN membership since 1971. Therefore, there was a long lag in obtaining the information regarding international anti-drug efforts. The essence and importance of implementing two UN drug conventions, i.e., the 1971 Convention on Psychotropic Substances, and the 1988 Convention against Illicit Traffic in Narcotic Drugs and Psychotropic Substances, were not known until the methamphetamine epidemic appeared in the early 1990s. As a result, the “Act for Prevention and Control of Illicit Drug Hazard” was eventually enacted in 1998 to fully comply with the three U.N. anti-drug Conventions [29]. But by the time when Taiwan implemented necessary control measures, illegal methamphetamine use has become a serious problem. It was thus a vivid example on the importance of international collaboration to conduct anti-drug efforts.

In Table 5, drug-related legislations were compared between Taiwan and Korea. The use of illicit drugs is regarded as a serious criminal offense (e.g., felony) in both countries under these legislations. Smuggling or manufacturing of illicit drugs can be punished by death penalty or life imprisonment in Taiwan, whereas it can be punished by imprisonment for life sentence or not less than five years in Korea.

As described previously, the Korean government has implemented a temporary scheduling system to the Act on the Control of Narcotics since 2011 [24, 29]. The new drug policy may have resulted in a surge of listed NPS items in Korea since then.

## Discussion

This study aimed at comparing the situation and trend of illegal drug use, with a focus on methamphetamine and NPS, between Taiwan and Korea from 2006 through 2014. Based on similar levels of demographic and economic status, the illegal drug use status between Taiwan and Korea was compared. While methamphetamine was confirmed to remain as the predominant drug in both Taiwan and Korea for decades, different illegal drug use patterns have been found in these two countries. With the *per capita* illicit drug seizures (Figs. 1, 2 and 3) and drug-related arrestees (most of them were illegal drug users) (Figs. 4, 5 and 6) for comparison, illegal drug use situation in Taiwan seems to be more worrisome than that in Korea. Taiwan is not a member state of the United Nations. Therefore, there has been no data regarding the illegal drug use situation in Taiwan reported to the U.N. or its affiliates. This study provides first-hand information of illegal drug use situation in Taiwan, especially on methamphetamine and NPS use, which can help complete the map of methamphetamine or ketamine flow in East and Southeast Asia [30].

In Korea, methamphetamine has been the most illegally used drug, followed by cannabis. However, a growing tendency has been noted toward the misuse of NPS or recreational drugs, such as synthetic cannabinoids, phenethylamines and propofol. In Taiwan, methamphetamine has also been one of the most illegally used drugs in addition to heroin, ketamine and MDMA. According to a previous study [13], ketamine replaced MDMA as the predominant drug in school-attending youths. The age of recreational drug (including ketamine) users was mostly under 27 years old. These adolescents were better educated, and the ratio of male to female was less than 3.5 [31].

The drug policy change may have an impact in illegal drug use and pattern. For example, heroin use by needle-sharing has been associated with HIV infection in the early 2000s in Taiwan [11]. Harm reduction policy, mainly with methadone maintenance treatment program and needle/syringe exchange program, was adopted in 2006 to curb the HIV spread among heroin injecting users [26]. The needle-sharing associated HIV spread has therefore been controlled. Although the harm reduction policy in Taiwan was originally implemented in response to the surge of HIV incidences among heroin injecting users, methadone maintenance treatment program of the harm reduction policy nevertheless helped the heroin addicts seeking treatments and may explain the fluctuation of heroin seizure and reduction of illegal heroin use from 2006 onward (Fig. 5). Since heroin use has not been a problem in Korea, harm reduction policy has not been adopted so far.

Regarding the NPS use, it was found that besides illegal ketamine use in Taiwan, synthetic cannabinoids, phenethylamines and propofol were popular in Korea (Table 4). Most NPS have not been scrutinized for their safety and may become a new threat to public health and security worldwide. For instance, it has been suggested that the unprotected sex due to NPS use would result in the spread of infectious diseases, especially HIV [32]. Some of the NPS have been scheduled and controlled. But still a lot of NPS remain elusive because listing of NPS in the UN or national schedules would require scientific evidence of drug dependence, abuse liability and ill health effects, which would be very difficult to collect data comprehensively without being officially scheduled. Rational scheduling of these NPS will pose a new challenge to incumbent anti-drug agencies. In contrast to the 23 items of NPS under control in Taiwan, the Korean government adopted the temporary scheduling system in 2011 and has resulted in a quick increase in the controlled items of NPS. Thus, the temporary scheduling system may provide a solution for emergency control while earning some time for research on the ill effects of a new item of NPS.

Other NPS of natural origin have also been identified. For example, Salvia has been available from the internet in Taiwan while Kratom has been confiscated in Korea [8]. Both have not been listed as controlled substances by the United Nations Conventions. Illegal drug use is a criminal offense in both Taiwan and Korea (Table 5).

While DLE plays a key role to remove drugs and high-risk offenders from the community, the most critical factor is whether a community is less burdened by the impact of drugs, such as crime, illness, injury and death in the longer term [23]. In addition, the illegal status of drug users may deter them from seeking treatment. Therefore, the outcomes of such drug policy may need further evaluation.

Different illicit drug patterns, as shown in this study, were observed in Taiwan and Korea even though these two countries are geographically adjacent and culturally similar. The results clearly indicate that geographical proximity could not serve as a sole determinant for the prevalence of illegal drug use. The aftermath of methamphetamine still lingers and harsh punishment may not be the only solution to curb the problem. The emerging issue of NPS, without a mechanism of early detection and scrutinizing the ill effects, would be difficult for further evaluation. A temporary or emergency scheduling may be imperative to identify and evaluate the potential problem of each individual NPS item.

## Limitations

Illegal drug use is a complicated neuro-psycho-social problem that intertwines with many individual, family and social factors. Therefore, evaluation of the drug use situation and pattern has never been easy. This is especially true when a comparison on the drug use situation is conducted between or among countries. A set of comprehensive data obtained in one country may not be used for comparison with the other country where such a data is unavailable. This was indeed the case when we performed this study. (For instance, for the purpose of demand side assessment, in Taiwan it was feasible to obtain the blanket data of drug urine test and admission for addiction treatment from TFDA while in Korea the data was confidential and not available. As a result, it was difficult to have comprehensive data for comparison on the demand side of illegal drug use between Taiwan and Korea.)

Nevertheless, we tried to use the data of drug-related arrestees instead because the behavior of illegal drug use, which is regarded as a criminal offense in both countries, may represent a proportion of demand side. 72.7 % and 55.8 % of arrests are illegal drug users in Taiwan and Korea, respectively (Fig. 4). The other limitation is the listing and control of NPS depends on the detection capacity and capability of the laboratories and efficiency of legislative or administrative process that could not be evaluated in this study. Even with these limitations, this study has depicted the difference in scale and pattern of illegal drug use between Taiwan and Korea for reference of action plan and policy-making.

## Conclusion

This study compared the illicit drug situation between Taiwan and Korea. In both Taiwan and Korea, methamphetamine has been the common problem, but illegal use of ketamine and heroin, which has been a major problem in Taiwan, has seldom been reported in Korea. In brief, the major illegal drugs were methamphetamine, heroin, and ketamine in Taiwan, whereas those were methamphetamine and cannabis in Korea. The NPS are emerging as a new threat but with different patterns in both countries. In Taiwan, ketamine and synthetic cathinones were the major categories while synthetic cannabinoids, phenethylamines and propofol in Korea. In terms of *per capita* illegal drug users, the illegal drug use situation in Taiwan is at a higher stake than that in Korea. Therefore, geographical proximity alone could not explain the different illegal drug use pattern between Korea and Taiwan. Further research on the policy change, Factors other than geographical proximity, such as drug policy and availability and accessibility to drugs will be important for further research.
